# First-year evaluation of a campus-wide, cross-disciplinary scholarly writing development program supported by a center for biomedical research excellence (COBRE)

**DOI:** 10.1371/journal.pone.0312322

**Published:** 2024-10-29

**Authors:** Amy M. Franks, Benjamin S. Teeter, Payton Davis, Mallory Allred, Reid D. Landes, Igor Koturbash, Judith Weber

**Affiliations:** 1 Department of Pharmacy Practice, College of Pharmacy, University of Arkansas for Medical Sciences, Little Rock, Arkansas, United States of America; 2 Department of Pediatrics, College of Medicine, University of Arkansas for Medical Sciences, Little Rock, Arkansas, United States of America; 3 Department of Biostatistics, College of Medicine, University of Arkansas Medical Sciences, Little Rock, Arkansas, United States of America; 4 Department of Environmental Health Sciences, Fay W. Boozman College of Public Health, University of Arkansas for Medical Sciences, Little Rock, Arkansas, United States of America; 5 Science Department, College of Nursing, University of Arkansas for Medical Sciences, Little Rock, Arkansas, United States of America; 6 Arkansas Children’s Research Institute, Little Rock, Arkansas, United States of America; University of the Witwatersrand Johannesburg Faculty of Health Sciences, SOUTH AFRICA

## Abstract

**Background:**

Scholarly publications are important indicators of research productivity and investigator development in Centers of Biomedical Research Excellence (COBREs). However, no information is available to describe implementation and evaluation of writing development programs within COBREs. Therefore, this paper aimed to evaluate the first year of a campus-wide COBRE-supported writing program.

**Methods:**

A convergent parallel mixed-methods design (QUAN + QUAL) was used. All writing program participants were invited to complete post-participation surveys, and a subgroup was selected using purposive sampling to complete individual semi-structured interviews. Descriptive statistics were used to characterize survey data, and qualitative content analysis was employed to analyze interview data. Self-determination theory served as the theoretical framework by which themes were developed and interpreted.

**Results:**

Professional staff, post-doctoral fellows, and faculty from all academic ranks (n = 29) participated in the writing program during its first year. Survey respondents (n = 18, response rate 62%) rated social support (89%), group accountability (89%), hearing group members’ writing goals (78%), receiving group advice (67%), and setting a weekly writing schedule (56%) as beneficial program components. Participants rated program benefits such as breaking away from other responsibilities, staying on task with writing goals, and receiving social support as most beneficial. During interviews, participants (n = 14) described five major themes related to the benefits received: 1) belonging to a community of writers; 2) managing writing-related emotions; 3) improved productivity; 4) establishing helpful writing habits; and 5) improved motivation for scholarly writing.

**Conclusions:**

This first-year programmatic evaluation demonstrates the writing program’s effectiveness as a campus-level development resource supported by a research center. Both survey and interview data affirmed that participants perceived autonomy, competence, and relatedness were supported through participation in the writing program. Participants placed particular emphasis on the writing program’s successful development of a community of scholarly writers.

## Introduction

The National Institutes of Health-funded Centers of Biomedical Research Excellence (COBREs) offer institutions within Institutional Development Award (IDeA) states three phases of multi-year support to advance the development and sustainability of thematic multidisciplinary research centers. Previous evaluations of COBREs have underscored the value of publications as indicators of faculty research advancement [1–4]. In a 2023 comprehensive evaluation of funded COBREs, Schaller found that COBRE investigators had published over 30,000 papers since the COBRE program’s initiation in 2000, with a median of 130 papers published per COBRE [3]. However, despite this collective success of COBREs, faculty of all academic ranks encounter barriers to writing that impede their scholarly productivity. Mentors from one COBRE’s evaluation identified scientific publications as the area in which junior investigators had made the least progress [1].

One COBRE, the Center for Childhood Obesity Prevention (CCOP), is highly committed to the professional development of the center’s faculty and staff members, and its scholarly writing program is central to this philosophy of developing and advancing its personnel. The CCOP’s writing program launched in fall 2021 as an adaptation of a program previously developed by one of the authors (AMF) for faculty within a single department at the same university [5]. The writing program was designed using the framework of self-determination theory (SDT) to support faculty and staff members’ needs of autonomy, competence, and relatedness in the area of scholarly writing [6]. The program’s three major components are described in Table 1. These components and their individual activities were intentionally developed to enhance intrinsic motivation for scholarly writing by supporting fulfillment of the three basic psychological needs associated with SDT.

**Table 1:** Description of writing program components.

Writing program component	Description of event/activities	Participants	Duration of Participation
**Write and Recharge social writing sessions**	Participants share a physical location while working independently on writing projects Held in on-campus conference rooms with videoconference connections when needed for distant-site participants Participate in goal setting and progress reporting Provide refreshments and lunch Includes a brief facilitated wellness session (e.g., mindfulness activity, chair yoga, etc.)	Limited to 25 per session CCOP-affiliated individuals are given first opportunity to participate, then registration is opened campus wide	5 hours
**Writer’s Block longitudinal writing development program**	Longitudinal cohort of scholarly writers who prioritize one or more writing projects for submission by the end of the program Schedule and complete regular writing time (at least weekly) Report total word count on shared spreadsheet each week Monthly virtual group session with reporting of writing progress and goal setting Share collective wisdom and experiences related to writing	Limited to 20 per cohort CCOP-affiliated individuals are given first opportunity to participate, then registration is opened campus wide	6 months, with a target submission date for writing projects at the end of the cohort period
**Off-campus overnight writing retreat**	Focused, protected writing time alongside other writers Held at a conference center that offers meeting space, overnight lodging, meals, and physical activities 1.5- to 3-hour writing session increments with participants sharing goals and progress for each session Share meals and participate in social activities	Participants of other components of writing program (Writer’s Block, Write and Recharge) are invited to attend	2.5 days

Initially, the writing program was designed to support CCOP-funded junior faculty as part of the stated COBRE goal of enhancing faculty development towards funding independence. However, over time, as the program’s popularity grew, the opportunity to participate was expanded to include faculty and staff in any program or college at the University of Arkansas for Medical Sciences (UAMS) as well those affiliated with the Arkansas Children’s Research Institute, thereby achieving campus-wide reach. As a result, the CCOP now offers a multi-component, cross-disciplinary, campus-wide program to support scholarly writing that is based on SDT. Few studies have evaluated the impact of a writing program through the theoretical lens of SDT. In this paper, we describe the early programmatic evaluation of this CCOP-supported, campus-wide writing program. Our mixed-methods analysis provides insight about how the basic psychological needs of autonomy, competence, and relatedness were supported by the program and how the program further supported their scholarly writing.

## Methods

We evaluated the process, structure, and initial offering of the CCOP writing program using a convergent parallel mixed-methods design (QUAN + QUAL) [7]. The quantitative phase used post-participation surveys, and the qualitative phase employed semi-structured interviews. Results from each phase were analyzed and interpreted to explain and triangulate the perspectives of writing program participants. The University of Arkansas for Medical Sciences institutional review board determined this program evaluation did not involve human research and waived the requirement for consent. All interview participants agreed to audio recording of the interview.

### Study setting and study population

The programmatic evaluation was conducted at the end of the first year of the CCOP writing program. Demographic characteristics of writing program participants were obtained from data collected during the program’s registration process. These demographic data were used to characterize the study population as shown in Table 2.

**Table 2:** Demographic characteristics of writing program participants and program evaluation participants.

	Writing program participants	Program evaluation survey participants^a^	Program evaluation interview participants
**Number of participants**	29	18	14
**Academic Rank/Role of Participants, n (%)**			
**Assistant professor**	14 (48)	6 (33)	9 (64)
**Associate professor**	5 (17)	5 (28)	3 (21)
**Professor**	5 (17)	5 (28)	1 (7)
**Professional staff member**	4 (14)	2 (11)	1 (7)
**Post-doctoral fellow**	1 (3)	0 (0)	0 (0)
**Academic Unit of Participants, n (%)**			
**College of Medicine**	16 (55)		7 (50)
**College of Nursing**	7 (24)		4 (29)
**College of Pharmacy**	2 (7)		1 (7)
**College of Public Health**	3 (10)		1 (7)
**Other**^a^	1 (3)		1 (7)
**CCOP affiliation** ^b^ **, n (%)**	13 (45)		6 (43)

^a^ Another university within the university system.

^b^ CCOP affiliation defined as receiving salary support, project funding, or having formal leadership or administrative role in the CCOP.

All participants of the Writer’s Block longitudinal program and/or the multi-day, off-campus writing retreat (n = 29) were invited to complete the post-participation survey(s) for the writing program component(s) they attended. Following the surveys, a purposive sampling approach was used to invite a subgroup of writing program participants to complete individual semi-structured interviews. Purposive sampling ensured participation by a representative sample of participants across writing program components, colleges, faculty ranks, and roles.

### Quantitative evaluation

Post-participation survey data were collected following the completion of the Writer’s Block longitudinal program and the off-campus writing retreat. Participants were invited to complete survey(s) via email invitation. Survey data were collected using REDCap electronic data capture tools hosted at the University of Arkansas for Medical Sciences [8,9].

Survey items were developed by the authors to elicit information from the participants’ perspectives about their participation in the writing program (S1 File). Survey instruments explored different aspects of satisfaction and productivity associated with writing program participation and were not pilot tested in advance. Survey instruments included independent Likert items, ordered response items, and dichotomous response items (yes/no). For the Writer’s Block longitudinal program post-participation survey, there were 16 Likert items (what resulted from participation in program, satisfaction with program), 3 ordered-response items (writing habits, experience level as a scholarly writer), and 11 dichotomous response items (recommend program, identify helpful aspects of program, identify productivity resulting from program participation). For the writing retreat post-participation survey, there were 18 Likert items (what resulted from participation in retreat, satisfaction with retreat), 1 ordered-response item (experience level as a scholarly writer), and 8 dichotomous response items (participate again, identify new collaborators, identify productivity resulting from retreat participation). For both survey instruments, Likert items included 5 response options (1 = strongly disagree to 5 = strongly agree or 1 = very dissatisfied to 5 = very satisfied). Survey data were downloaded to a Microsoft Excel spreadsheet for review and analysis. Descriptive statistics were used to characterize the data. Likert items were analyzed independently using descriptive statistics (median, frequency, percent) and not analyzed as an aggregated or summed scale. Ordered response and dichotomous response items were also analyzed using descriptive statistics (frequency, percent).

### Qualitative evaluation

Semi-structured interviews were performed to collect data for the qualitative component of the evaluation. One author (BST), who was experienced in qualitative methods and not involved in the design or facilitation of in the writing program, conducted in-person or videoconference interviews with individual participants. All interviews took place within one month of the individual’s participation in the writing program.

The interview guide (S2 File) was developed by the authors using concepts deemed important to the design of the writing program. Using open-ended prompts, the interviewer solicited information about each participant’s experiences in the writing program, focusing on which elements of the program were most beneficial and how the program benefitted participants. Questions were developed *a priori* to probe participants’ perspectives as well as generated in response to participants’ previous answers to elicit greater detail. Interviews were continued until data saturation was reached. Interviews were digitally recorded, transcribed verbatim by a professional transcriptionist, and checked for accuracy by one of the authors. Interview transcripts were de-identified and uploaded to MaxQDA 2020 (VERBI Software GmbH, Berlin, Germany) for analysis using a qualitative content analysis approach.

Two authors experienced in qualitative analysis developed a coding scheme using both inductive and deductive approaches (S3 File). The initial coding scheme included codes derived from the writing program components and activities, interview guide, and literature review. This coding scheme was further refined by a run-in review of interview transcripts by two authors to identify new concepts not included on the initial coding scheme. Codes and subcodes centered around three major categories: 1) activities and characteristics of the writing program that were beneficial, 2) the benefits experienced by writing program participants, and 3) suggestions for how the writing program could be improved. The coding scheme was entered into a MaxQDA project file, and all subsequent coding was performed using this software. Two authors independently coded each transcript using a constant comparison approach to ensure coding agreement, increase trustworthiness, and minimize bias. Discrepancies in coding were resolved by review of coded segments and discussion between coders. When needed, a third author assisted in reaching agreement between coders. After coding was completed, codes were grouped into categories, and themes were developed and interpreted drawing from the framework of SDT. Code frequencies were generated to further describe trends in qualitative data. Considerations according to the Consolidated Criteria for Reporting Qualitative Research (COREQ) [10] were used to guide the evaluation and preparation of this work (S4 File).

## Results

Eighteen of the 29 writing program participants completed post-participation survey(s), yielding a response rate of 62%. Survey participants were broadly representative of all participants in the writing program according to position/faculty rank. Table 2 provides demographic information about participants completing post-participation survey(s).

**Fig 1:**
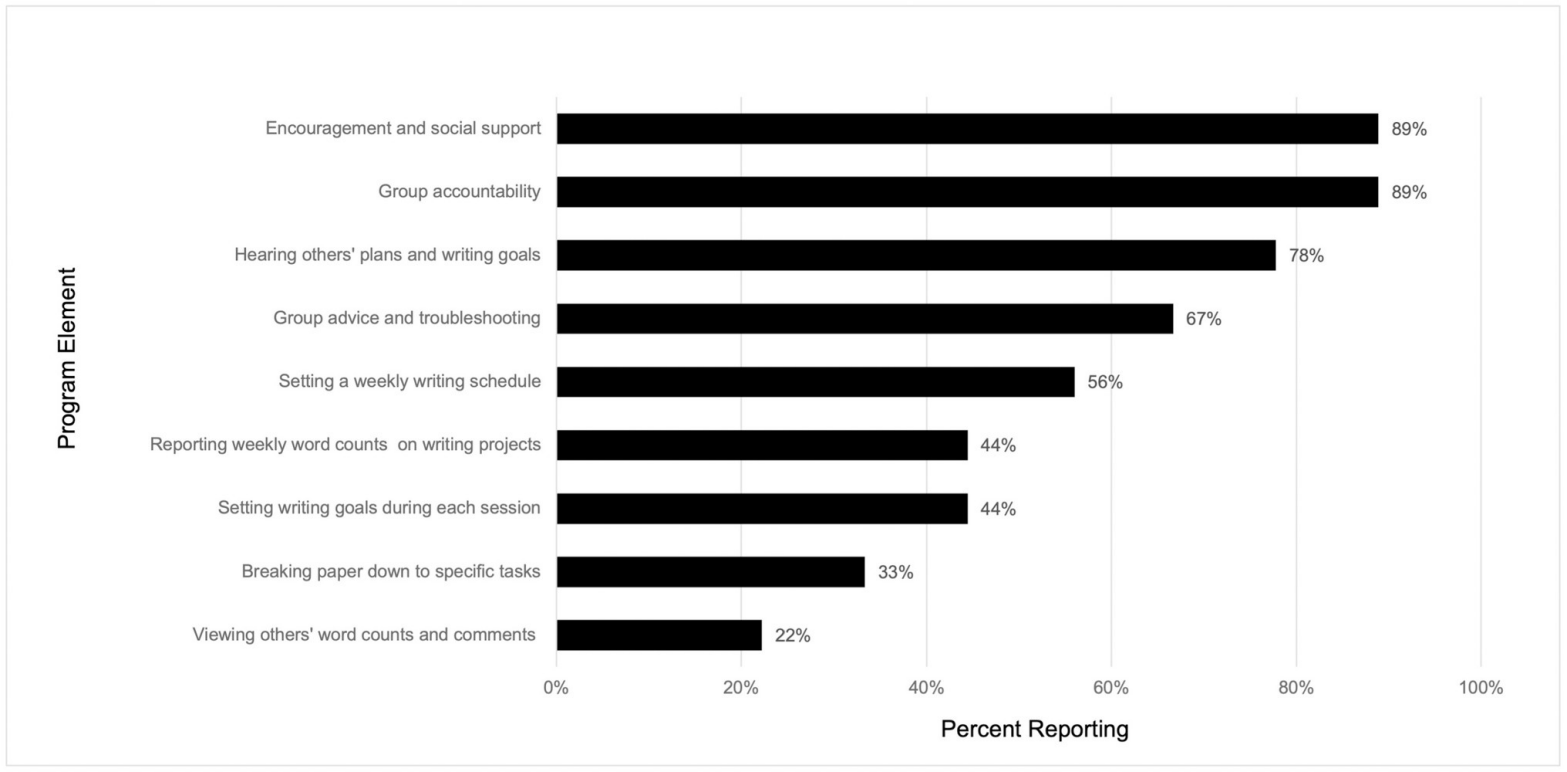
Percent of Writer’s Block longitudinal program participants (n = 9) reporting individual program elements as beneficial on post-participation survey. Response to survey item “What aspect(s) of [writing program] was/were helpful to you? Please choose one or more options”.

**Fig 2:**
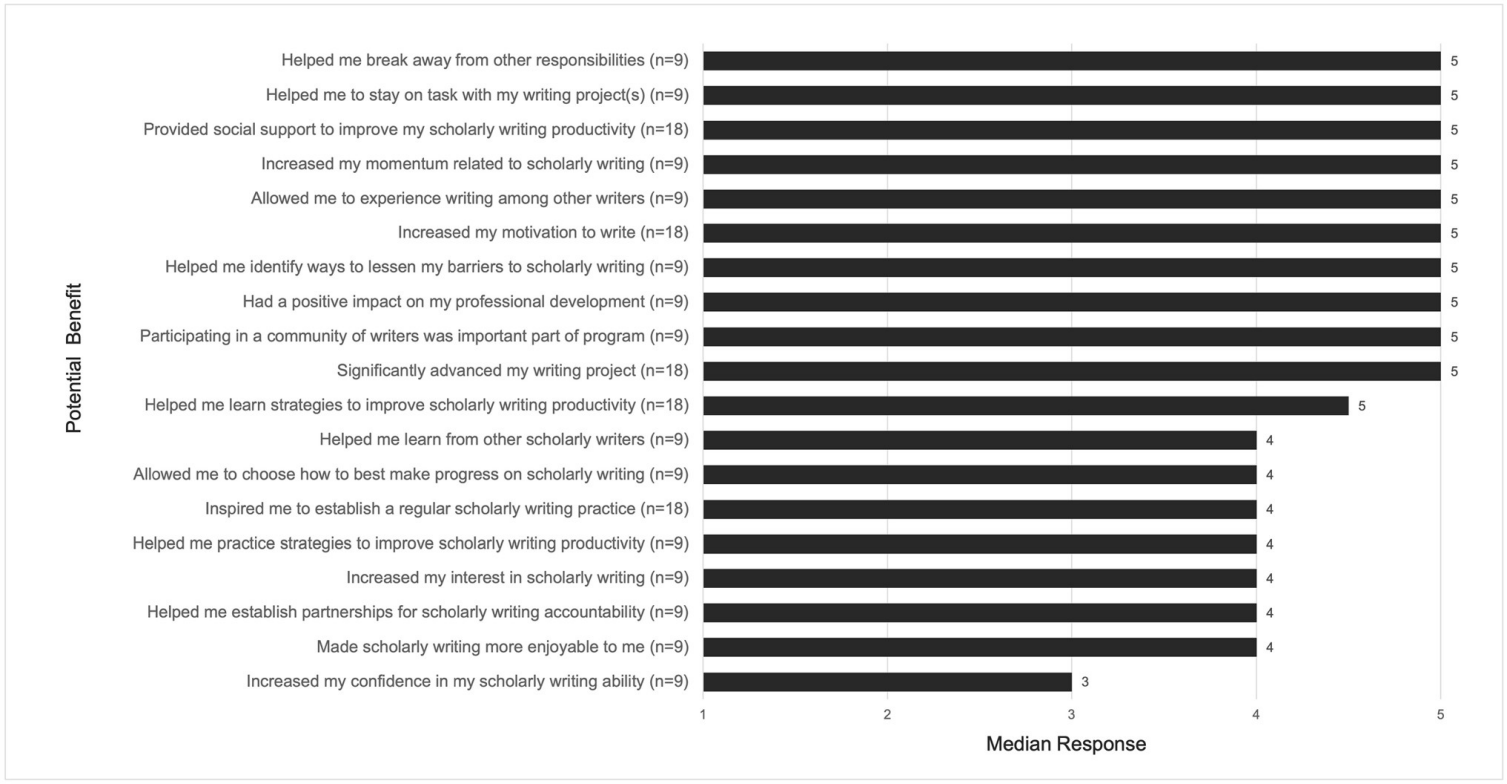
Median responses on post-participation survey of 18 potential benefits resulting from writing program participation. Response to the Likert item “Please report your agreement with each statement about [the writing program] below using a scale of 1 to 5, with 1 = strongly disagree and 5 = strongly agree. The [writing program]…”.

All survey respondents agreed that they would participate in the writing program again or recommend the program to others (n = 18, 100%). Survey responses affirmed that the writing program supported the three basic needs of autonomy, competence, and relatedness (Figs 1 and 2). The top four most highly rated writing program elements from the surveys specifically supported relatedness through group interactions, thus *highlighting the value of developing community* with other scholarly writers (Fig 1). Survey results also indicated wide-ranging benefits resulting from participating in the writing program. Agreement with specific program benefits is depicted in Fig 2. Collectively, survey respondents indicated that the program helped them manage their own writing habits, increase their motivation for and productivity in writing, and develop a community of scholarly writers.

Fourteen writing program participants completed individual semi-structured interviews, with interviews lasting an average of ~28 minutes (range 17–39 minutes). Demographic characteristics of interview participants were similar to those of all writing program participants and are shown in Table 2.

The five program elements most frequently described as beneficial by interview participants were submitting word counts (n = 12, 86%), having dedicated writing time with others (n = 11, 79%), listening to other participants’ successes and challenges (n = 11, 79%), setting monthly writing goals (n = 10, 71%), and reducing distractions during social writing sessions (n = 10, 71%) (Table 3). It was evident that participants valued the program elements that helped them self-regulate, since setting goals, submitting evidence of progress (word counts), and limiting distractions all enhanced participants’ self-reported writing productivity. These program elements contributed to fulfilling the need for competence and autonomy. Participants’ discussion of activities that were social in nature, such as writing with others and engaging in discussions with fellow participants during writing group meetings, were strong indicators of the writing program’s support for relatedness.

**Table 3:** Top five writing program elements reported as beneficial during semi-structured interviews of writing program participants.

Writing program element or characteristic	Psychological need supported	Number (%) of participants reporting (n = 14)	Number (%) of mentions (n = 383 coded segments)	Example quotations
**Submitting word counts**	Competence	12 (86)	36 (9)	“I think it [submitting word counts] helped. It was very straightforward, I opened the document, looked…word count at the bottom, I typed in that number and it went up, it went down, but, and then we could make comments at the bottom like, you know, I was writing so I added a lot and then, you know, I was editing, I cut out a lot, both are types of progress and the word count would go in the opposite direction.” (Participant 12) “…for me it helped because, 1) …it kept [my three major items] at the forefront of my mind, that these are the things that you’ve prioritized for yourself, get them done. And then 2) I’m also showing people my progress so it’s that accountability factor where they see how far or how little I’ve come, and then if I’m struggling then that’s something we can talk about during the monthly meeting.” (Participant 13)
**Dedicated, focused writing time with others**	Relatedness	11 (79)	58 (15)	“I think it’s the exclusive environment, like everyone who’s there is there because they’re wanting to write and we’ve all cleared our calendars to do it, is what makes it work. But yet if you were to be put in a little room by yourself it wouldn’t feel the same, it would be more distracting…it’s just, the environment is such that everybody’s here writing, I’m going to write, you know. I don’t know how else to explain it.” (Participant 4) “To have the time protected, so that was a huge deal for us. You know, so nobody bothered anybody because they knew this is sacrosanct, this is like really important to be here doing this.” (Participant 5)
**Hearing others’ successes and challenges during monthly meetings**	Competence, Relatedness	11 (79)	27 (7)	“Yes, it helped to know that I’m in a similar boat as other people and that some of these people that I even look up to, some of the more senior people in the group, hearing them talk about having similar issues, I guess kind of took some of the pressure off of myself that I’m putting on there, and some of that disappointment in myself.” (Participant 3) “I find it really useful for a multitude of reasons…you knew in a month there was a goal to meet, you have to talk about struggles, hear other people’s wins, which is always exciting, and is also like fuel for motivation.” (Participant 7)
**Setting goals for writing sessions between monthly meetings**	Competence, Autonomy	10 (71)	34 (9)	“I’m doing it for each of the sessions, just small, small, very small goals and small steps at the time to be able to achieve, that I haven’t been doing actually before, I think this, this is an innovation for me, I really like that, small steps and check to see whether you have achieved that goal at the end.” (Participant 1) “I’m going to work on this specific task for this hour and a half or two, whatever it is, and then you can do that. And so you kind of figure out how to make it a bite-sized piece that you can accomplish in a short amount of time.” (Participant 2)
**Reduced distractions while writing alongside others**	Relatedness, Autonomy	10 (71)	23 (6)	“Well, to be honest, actually I was a little hesitant to be able to write in between people, in the same room with the people because I always thought that I may get distracted when people move or [click] on the keyboard, but to my surprise it seems like a library environment there, [laughter] so I would consider doing it again in the future. But may I achieve the same level of concentration if I do it by myself? I don’t know that.” (Participant 1) “It’s literally the peer pressure of other people being grownups and being focused that keeps me from spending the whole time being like, oh, is that a red maple tree, you know, [laughter] like is that indigenous here, I’ll look it up, you know.” (Participant 8)

Table 4 shows themes related to the benefits received by interviewed participants. All participants (100%) reported a sense of connection and belonging with other writers as a benefit of participating in the writing program. This relatedness occurred despite participants working on separate writing projects. Within this theme, several participants spoke about psychological safety among group members, and many described increased energy for writing when participating in social writing. All interview participants (100%) also discussed positive changes in their emotions related to scholarly writing. Participants described increased enjoyment of the writing process as well as reduction in negative emotions such as a fear and anxiety about their writing. Most (86%) also described improvement in their writing productivity as a result of their participation, thus realizing the progress they had made toward their writing projects or a reinvigoration for writing. Eleven (79%) participants described the development of new habits as a benefit to their participation in the writing program, and these new habits contributed to greater enjoyment and/or productivity in writing. Comments from nine participants (64%) also pointed to improved motivation to engage in scholarly writing, including placing a higher priority on writing as a responsibility of their work.

**Table 4:** Benefits resulting from participation in the writing program as discussed during semi-structured interviews.

Theme	Definition	Number (%) of participants reporting (n = 14)	Number (%) of mentions (n = 259 coded segments)	Example quotation (participant number)
**Belonging to a community of writers**	Participants experienced relatedness with others through a sense of like-mindedness toward writing, shared research interests, and/or psychological safety during group discussions	14 (100)	93 (36)	“It goes a little beyond just the social interaction, I think [facilitator] did an amazing job at engaging certain senior level faculty who made it feel safe, who weren’t like big and scary, because we’re used to dealing with big, scary, intimidating, full professor, tenured faculty every single day who don’t, who may not be as supportive, encouraging, who may not understand and identify with the failures that we’re experiencing during our writing processes…I kind of got the sense that it was intentional, that she engaged them into being a part of Writer’s Block for the purpose of helping set the tone, that very supportive tone, that safe tone…and we never had a fear of anything leaking out of Writer’s Block.” (Participant 14) “I participated in the Write and Recharge, loved, loved, loved it, and found out I really enjoy parallel writing, I like being in a group with people who are like-minded, so the energy is real positive and they’re really in the zone. Nobody bothers you, you’re doing what you need to do, and it was great.” (Participant 5)
**Managing emotions related to writing**	Participants experienced enhanced positive emotions (e.g., enjoyment, satisfaction) and alleviated negative emotions (e.g., anxiety, disappointment) toward writing	14 (100)	43 (17)	“I was never taught how to write for manuscript purposes…I never had a mentor to really show me how to write or how to dedicate that time or, or any of that. It’s been a struggle, and honestly, it’s been terrifying. So this has been really a wonderful way to like demystify the process a little bit.” (Participant 5) “Dealing with criticism of papers… was very difficult, to the point to where it was immobilizing…So Writer’s Block helped me deal with issues like that, literally, hey, team, this week I’m going to open up the email I got back and I’m going to review it, and then there was, it was such a supportive community, faculty at all levels participated in Writer’s Block and they would say, ‘Well, let me review it for you, let me break it down into different categories of importance and value and pass it along to you’…So I’m not as fearful of comments anymore.” (Participant 14)
**Improved productivity in scholarly writing**	Participants met goals, completed or made progress toward writing projects, and/or jumpstarted new writing projects	12 (86)	50 (19)	“There were two papers I wanted to get out, one I did; [the other] was not as far along as I thought it was but I’m still making progress on it. And then there’s actually a different one that I made more progress on because the results were further along than the other paper…I got [it] done that had been sitting there for like a few years. So getting some of those old papers revived and in for submission is huge.” (Participant 2) “It’s helpful in that, it’s that moment of reflection that I probably wouldn’t have otherwise, and there is something very reinforcing about, you know, realizing you’re making progress and kind of stopping and acknowledging it, you know.” (Participant 8)
**Establishing helpful writing habits**	Participants adopted practices that made writing more productive and/or enjoyable	11 (79)	49 (19)	“I mentioned that I would like a social writing partner, someone that would meet me somewhere and write because…I was really surprised at how much I wrote just sitting in a room full of people writing. And so I asked…if anyone was interested and [participant name] reached out and we’d meet every Tuesday morning…and so now I know Tuesday mornings from 9–11 is writing time and that’s been the most beneficial.” (Participant 3) “…the biggest win I have from this besides blocking time [for writing] and setting boundaries is managing what I can do in the time…I block this time and I protect it so if [colleagues] want to schedule this like silly [meeting name] in my writer’s block time that I set for myself, I know that it’s not going to make or break my career or change anything that I can’t read in the minutes.” (Participant 7)
**Improved motivation for scholarly writing**	Participants experienced increased desire or willingness to write	9 (64)	19 (7)	“I’m very much benefiting from [the writing program] because I think I need timelines. When I have timelines, when I know that I am expected to deliver an outcome at a certain date then I, I get more motivated, I try to carve out more time from my non-clinical duties basically, sometimes after hours, early in the mornings, on the weekends, because I was able to set my goals for a month, realistic expectations that I set for myself, that I was able to deliver that goal to the best of my ability.” (Participant 1) “So [participant name] was actually in my group, so hearing about some of his challenges, and he’s such a, a great researcher and so dedicated and writes like amazing things, so I was like if he’s having challenges and he can still like rock it and [laughter] do all these amazing things, I’m like, okay, I have no excuse, he’s busier than I am so I better get cracking and figure out how to do what he’s doing.” (Participant 2)

Interview participants provided suggestions for how the writing program may be improved (Table 5). Overall, participants reported being satisfied with the quality and benefits of the writing program, and suggestions for improvement varied considerably among interview participants. For example, one participant advocated for lengthening the Writer’s Block longitudinal program from a 6-month program to a year-round offering, while another recommended shortening the program to four months. Interview participants most commonly voiced support for offering more opportunities for social writing (n = 6, 43%) and enhancing the accountability measures of individuals (n = 6, 43%).

**Table 5:** Participant suggestions for writing program improvement.

Suggested improvement	Psychological need supported	Number (%) of participants reporting (n = 14)	Number (%) of mentions (n = 85 coded segments)	Example quotation
**Increase opportunities for social writing (writing among others)**	Relatedness	6 (43)	11 (13)	“I think I would have benefited from having, committed, protected time with other people, not just left to my own devices and discipline…I don’t no-show to meetings with other people but I no-show to meetings with myself all the time.” (Participant 8)
**Improve accountability methods**	Competence	6 (43)	12 (14)	“I would say if there are any ways to expand the word count component, we should certainly do it. I don’t know what that would look like…if there’s a way to have a more formal system that reminds you throughout the week if you haven’t logged the word count in three days or in seven days, that would be a really good feature.” (Participant 14)
**Incorporate changes to monthly group meetings**	Competence	5 (36)	6 (7)	“[if] we had a set of lectures… where we kind of planned out the papers, we tackled each, component of the paper, so introduction section, first methods…we wrote all of those up. I think it may be a good idea to expand those components of Writer’s Block.” (Participant 14)
**Increase social aspects of writing program**	Relatedness	4 (29)	6 (7)	“The social part, you know, getting to know other people either in your field or related fields and being able to bounce ideas off each other, that wasn’t expected until I started the program. We didn’t do a lot of it, though, we did some, I would have probably liked a little bit more.” (Participant 2)
**Change length of longitudinal writing program**	Competence	3 (21)	8 (9)	“I think it should span the entire year because…[writing] is a part of the job descriptions…so if we can expand it to yearly, I would participate in Writer’s Block every single year. Because now that it’s ended, I no longer have that accountability factor, the word counts that I’m logging, all the benefits of Writer’s Block…I’m just sort of still being productive because of the things that I learned in Writer’s Block.” (Participant 14)

## Discussion

In this early program evaluation, we focused on how the program was received by participants during its first year. Because the program was designed using an SDT framework, it was important to learn how the multidisciplinary group of participants viewed the program in terms of supporting their needs and how it benefited their scholarly writing. Data from survey responses and interviews show that participants viewed the program as supportive across all three basic psychological domains of autonomy, competence, and relatedness. The writing program was successfully expanded from a department-level approach within a single college to a cross-disciplinary campus program offered broadly to faculty and staff. The program represents a model for other institutions as a first step toward creating an institution-wide writing community.

The basic premise of SDT in an academic work context is that when the work environment helps faculty or staff members meet their basic psychological needs related to autonomy, competence, and relatedness, their intrinsic motivation to complete difficult self-directed tasks increases [6]. According to SDT, this should result in less work-related fatigue, a sense of value and ownership related to work responsibilities, greater enjoyment and engagement in writing, and enhanced motivation to pursue and complete scholarly writing projects [6,11,12]. Our evaluation demonstrated evidence of supporting all three basic psychological needs. The program’s emphasis on autonomy (prioritizing writing projects, setting writing goals, and instilling positive writing habits), competence (learning new writing-related skills, gaining writing experience, and developing confidence toward identity as a scholarly writer), and relatedness (discussing practices and progress with other writers, embracing writing as a social activity) equipped participants to adopt strategies to create and maintain regular progress toward publications. Participants valued opportunities to connect with others, demonstrating the program’s emphasis on a less traditional view of writing as a socially connected activity. As a result, participants reported experiencing a sense of belonging, enhanced self-regulation, and improved productivity. These are important lessons that help solidify the effectiveness of an SDT-based writing development program on instilling an institutional culture that supports scholarly writing.

We found only one other description of an SDT-based writing program in the published literature. Winnie and colleagues described a longitudinal writing support program for first-time physician authors who had previously presented their work at a national conference [13]. Similar to our findings, their evaluation of 9 participants’ comments indicated support of needs related to autonomy, competence, and relatedness. However, their program focused on the technical production of a manuscript through deadline-setting, peer feedback, and group discussion, which is in stark contrast to our program aimed at supporting scholarly writers through encouraging the establishment of productive writing habits and creating a supportive environment for engaging in social writing.

We are using the feedback received from this first-year evaluation to further enhance ongoing and future offerings of the writing program. Participants’ suggestions for further improving the program included more opportunities for social writing and additional focus on improving participants’ accountability to writing progress. In response, we are offering more social writing events and expanding opportunities for participants to learn more about each other and their work after receiving these suggestions. These suggestions highlight the participants’ need for relatedness and belonging to a community with similar interests, needs, and expectations for success. In addition, previous work supports enhancing accountability to the writing group. Individual commitment, and its effects on trust and development of relationships among group members, has been identified as a key factor for the success of a writing group [14]. Olszewska and colleagues highlighted the importance of fostering relational, communal, and institutional commitment and sustainability as writing programs are developed [14]. Grzybowski and colleagues found that faculty who frequently attended a writing group published more than those who infrequently attended or did not participate [15]. We believe increased accountability will not only enhance the individual’s experience in the program, but will also enhance the group’s interactions, sense of relatedness, and the overall sustainability of active participation in the program [14].

Our early evaluation provides insight on the acceptability and benefits of the writing program as perceived by its participants, as well as direction on how to further support the development of a writing community among faculty and staff at the institution. Although we have not yet evaluated the impact of the writing program on participants’ output of scholarly publications, the behaviors valued and demonstrated by participants suggest that writing habits have improved and may lead to an improvement in scholarly writing productivity. Ongoing evaluations of the writing program will consider the longer-term effects of the program on writing productivity, namely paper submissions and publications in peer-reviewed journals. While this initial evaluation also supports the expansion of a department-level development program to a campus-wide, multidisciplinary initiative, it is not yet known whether this is generalizable to other institutions or other types of support mechanisms. Finally, our data is limited by selection bias, since participants elected to enroll in the writing program and may have been more motivated to increase writing productivity than those who did not enroll in the program. Because the writing program emphasizes the development of intrinsic motivation by meeting basic psychological needs, we feel that mandatory participation would not be an effective strategy to broaden participation. However, helping potential participants such as junior investigators in COBREs better understand the goals and components of the writing program may encourage more participation by those who may not have otherwise enrolled in the program.

## Conclusions

We successfully demonstrated the expansion of a department-level scholarly writing faculty development program to a research center-supported, campus-wide, cross-disciplinary model for a diverse group of faculty and staff. This is unique in that the writing program leveraged the resources of a research center (the CCOP COBRE) to build a faculty development program open to faculty and staff from across all campus units. Participants perceived the writing program to be beneficial in supporting all three basic psychological needs described by SDT. Further, participants reported developing a sense of community, experiencing more positive emotions and motivation, and improving habits and productivity related to writing as a result of participating in the program.

## Supporting information

S1 File Post-participation surveys for writing program. (PDF)

S2 File Interview guide. (PDF)

S3 File Coding scheme. (PDF)

S4 File COREQ checklist. (PDF)
