# Supplementary material for: First-year evaluation of a campus-wide, cross-disciplinary scholarly writing development program supported by a center for biomedical research excellence (COBRE)
Source: PLoS One. 2024 Oct 29;19(10):e0312322. doi: 10.1371/journal.pone.0312322 (PMC11521313; doi:10.1371/journal.pone.0312322)
Supplement: S1 File — (PDF) [file pone.0312322.s001.pdf]

Writer's Block Post-Participation Survey

Thank you for your participation in the CCOP's Writer's Block 6-month writing development program! It is very important to us to provide a program that meets your needs and supports your scholarly writing. To help us learn what worked for you, please complete the survey below. All responses will be reported in aggregate only and no personal identifiers will be included.

Please limit your responses to your experiences as a participant in the Writer's Block 6-month longitudinal writing program and do not consider other components of the CCOP writing program (Write and Recharge, writing retreat, etc) in your responses.

1. Please report your agreement with each statement about Writer's Block below using a scale of 1 to 5, with 1=strongly disagree and 5=strongly agree.

Writer's Block…

	1 = strongly disagree	2 = disagree	3 = neutral	4 = agree	5 = strongly agree
…increased my interest in scholarly writing.					
…increased my motivation to write.					
…increased my confidence in my scholarly writing ability.					
…provided social support related to scholarly writing.					
…helped me learn from other scholarly writers.					
…helped me establish partnership for scholarly writing accountability.					
….helped me practice strategies to improve my scholarly writing productivity.					
…inspired me to establish a regular scholarly writing practice.					
…made scholarly writing more enjoyable to me.					
…had a positive impact on my professional development.					
…helped me to significantly advanced my writing project(s).					
…was worth my time.					
…should continue to be offered.					

2. Please consider the following statements and report your agreement with each on a scale of 1 to 5, with 1=strongly disagree and 5=strongly agree.

	1 = strongly disagree	2 = disagree	3 = neutral	4 = agree	5 = strongly agree
Participating as part of a community of writers was an important part of Writer's Block.					
My momentum related to scholarly writing increased as a result of my participation in Writer's Block.					

3. Which best describes your current scholarly writing habits?
   I do not have a regular writing time / I write only when I feel I have time.
   I work on my writing project once monthly.
   I work on my writing project several times a month but not at scheduled intervals.
   I write every week.
   I write multiple times every week.
   I write every day.

4. On average, what is the duration of your current scholarly writing session?
   Less than 30 minutes
   30-59 minutes
   60-119 minutes
   120 minutes or more
   Not sure

5. Which best describes your experience level as an author of scholarly writing?
   Novice
   Intermediate
   Advanced
   Expert
   Not sure

6. What aspect(s) of Writer's Block was/were helpful to you? Please choose one or more options.
   Setting a personal weekly writing schedule
   Setting writing goals during each session
   Breaking paper down to specific tasks
   Reporting weekly word counts and other progress on writing project(s)
   Viewing others' word counts and comments on shared file
   Group accountability
   Formal peer reviews conducted by another group member
   Group advice and troubleshooting
   Hearing others' plans and writing goals
   Encouragement and social support
   Other (please specify): [open response]

7. Will you recommend Writer's Block to other faculty?
   Yes
   No

8. What did you like about Writer's Block (if not already noted in a previous question)? [open response]

9. How could Writer's Block be improved? [open response]

CCOP Writing Retreat Post-Participation Survey

Thank you for participating in our CCOP Writing Retreat! We hope you found your participation in the retreat to be rewarding. To help us further improve the experience, please complete the following survey items. All responses will be reported in aggregate only and no personal identifiers will be included.

Please limit your responses to your experiences at the Writing Retreat and do not consider other components of the CCOP writing program (Writer's Block, Write and Recharge, etc) in your responses.

1. Please report your agreement with each statement using a scale of 1 to 5, with 1=strongly disagree and 5=strongly agree.

   My participation in the CCOP Writing Retreat…

	1 = strongly disagree	2 = disagree	3 = neutral	4 = agree	5 = strongly agree
…helped me stay on task with my writing project(s).					
…allowed me to experience writing among other writers.					
…helped me learn strategies to improve my scholarly writing productivity.					
…allowed me to choose how to best make progress on my scholarly writing.					
…helped me break away from other responsibilities.					
…inspired me to establish a regular scholarly writing practice following the retreat.					
….helped me identify ways to lessen my barriers to scholarly writing.					
…increased my motivation to write.					
…helped me to significantly advance my writing project(s).					
…provided social support to improve my scholarly writing productivity.					

2. Did you identify any new potential collaborators or writing partners from your interactions at the Writing Retreat?
   Yes
   No
   Not sure

3. Please rate your satisfaction with the below aspects of the retreat on a scale of 1 to 5, with 1= Very Dissatisfied and 5= Very Satisfied.

	1 = very dissatisfied	2 = somewhat dissatisfied	3 = neutral	4 = somewhat satisfied	5 = very satisfied
The indoor environment at the retreat center.					
The outdoor environment at the retreat					

center.					
Social times to interact with other participants (activities, meals, breaks, etc.)					
The Q&A discussion with the panel of associate editors (Zoom session).					
The overall amount of time spent working on your writing projects.					
The pre-writing activities for each writing session (identify objectives for writing session, log word counts).					
The post-writing activities for each writing session (assessing objective achievement, log word counts, rating satisfaction with progress).					
Amy Franks' role as the retreat facilitator.					

4. If given the opportunity, would you attend this Writing Retreat again?
   Yes
   No
   Not sure

5. Please describe how the Writing Retreat helped facilitate your scholarly writing. [open response]

6. Please suggest how future Writing Retreats might be improved. [open response]

7. Please consider the one or more writing projects you worked on during the writing retreat. Which statement(s) describe your progress during the retreat? Please select all that apply.
   I started a new writing project at the writing retreat.
   I added new content to an existing writing project at the writing retreat.
   I edited another author's contributions to an existing writing project at the writing retreat.
   I finished a complete draft of a writing project to send to coauthors, mentors, or colleagues willing to review.
   I incorporated another author's or reviewer's suggestions to an existing writing project at the writing retreat.
   I submitted a writing project to a journal, editor, or other external body.
   Other (please describe): [open response]

8. Which best describes your experience level as an author of scholarly writing for publication (manuscripts, book chapters, etc)?
   Novice
   Intermediate
   Advanced
   Expert
   Not sure
