# Supplementary material for: First-year evaluation of a campus-wide, cross-disciplinary scholarly writing development program supported by a center for biomedical research excellence (COBRE)
Source: PLoS One. 2024 Oct 29;19(10):e0312322. doi: 10.1371/journal.pone.0312322 (PMC11521313; doi:10.1371/journal.pone.0312322)
Supplement: S2 File — (PDF) [file pone.0312322.s002.pdf]

**Introduction**

We are conducting interviews with individuals that participated in Writer's Block, Write & Recharge, and/or the Writing Retreat as part of an overall evaluation of the writing program. During the interview I will ask you general questions about your writing and specific questions about program components. I would also like to hear about your general thoughts about the program overall.

- What would you consider barriers to writing?
  - Did participation in Writer's Block and/or the retreat help address those barriers? Which ones?
- How do you feel when you're writing scholarly works? Do you find it enjoyable? Frustrating?
  - I have this idea of being "nerd famous" that has a lot to do with being recognized as a leader in my field and having my publications cited, grants awarded, etc. Do you write to make a mark and be recognized in your field? Do you write to make sure others know about your important findings?
    - How do I ask about writing even if no one will read it……..? Would you try to publish your findings even if you thought no one would read them? Do you write and publish work when the findings aren't what you had hoped?
- My list of program participants indicates you participated in _______________(WB, W&R, R). I know they are all pieces of a bigger program but for this interview I'd like you to try to think about them as separate programs.

**Writer's Block Questions**

- When you registered for Writer's Block, what were your expectations for the program?
  - Ideas about the kind of activities you would be asked to do?
  - Thoughts about the time commitment or frequency of meeting?
- Did Writer's Block align with your expectations? Why or why not?
- What do you think was most beneficial about Writer's Block? Why?
  - Prompts: setting writing goal, discussing goals and progress with other participants, setting a regular writing schedule (at least weekly), target date for manuscript submission, etc.
- A big part of Writer's Block is the social interaction with peers. Can you tell me about that?
  - What did you like about discussing your goals, progress, sharing writing tips, etc.?
  - Closeness of the group?
  - Comfortable giving and receiving feedback from peers?
- What did you think about the shared spreadsheet where you reported your word count and writing progress?
  - Like it? Dislike it?
- Did you have a writing group you received support and feedback from prior to enrolling in Writer's Block?
  - If yes, what did that look like? A mentor? Team writing?
  - If not, is that something you'd like to continue?
- Are there any other specific activities you were asked to do in Writer's Block that you would like to continue now that Writer's Block has ended?
  - Word counts, setting a regular schedule, etc.
- What would you change or add to Writer's Block that would improve the program?
- Would you want to participate in Writer's Block again? Why or why not?
- Have you participated in other longitudinal writing workshops?
  - What do you think about Writer's Block in comparison to other writing groups/workshops?
- What did you think about the length of the program?
  - Is 6 months too long? Too short?
- What about meeting frequency, word count frequency?

**Write & Recharge Questions**

Now I'd like to ask you a few questions about Write & Recharge. Again, try to think about this as separate from the other programs.

- When you signed up for Write & Recharge, what were you expecting to get out of the half-day event?
- What about Write & Recharge was most beneficial for you?
  - Protected writing time, writing alongside others, social interactions, sharing topics and progress with peers, wellness activities, getting away from the office to a new setting free from distractions, etc.
- What did you think about setting a writing goal for such a short period of time?
  - How was it helpful/not? Do you remember what your goal was? Did you reach your goal?
- Was writing alongside others something you had done previously? What do you think about it?
- Did you know others at the event? Any new connections made during the event?
- What do you think would improve Write & Recharge?
- Would you make plans to participate in Write & Recharge if offered again? Why or why not?
  - Would you schedule your own writing afternoon away from the office with peers to continue writing progress?

**Writing Retreat Questions**

Now I'd like to ask you a few questions about the Writing Retreat. Again, try to think about this as separate from the other programs.

- When you signed up for the Writing Retreat, what were you expecting to get out of the 3-day intensive retreat? Why did you choose to participate?
- What are the most beneficial elements of the retreat?
  - Protected writing time, celebrating success/progress, writing alongside others, social interactions, sharing topics and progress with peers, wellness activities, getting away from the office to a new setting free from distractions, etc.
    - Specific stuff: Spill your brain before writing, specific list of things that need to get done during a specific writing session, number or words you currently have and what you want to have done by the end of the writing session…
- What do you think about getting away from home and the office to work on scholarly writing? Is it something you have done before?
- What have you learned about your scholarly writing habits/abilities at this retreat?
- What do you think about sharing your progress and struggles with peers?
- What did you think about the unwinding time?
  - Drinks in the lodge with discussion; Group hike; Firepit/smores?
- What changes do you think would improve the Writing Retreat?
- Would you make plans to attend another Writing Retreat if offered again? Why or why not?
  - Would you schedule your own retreat away from the office with peers to continue writing progress?
- What goals did you have for the writing retreat?
  - Have you achieved them? Or are you on your way to achieving them?
- Was writing alongside others something you had done previously? What do you think about it?
- Did you know others at the retreat? Any new connections made during the event?
