# Supplementary material for: First-year evaluation of a campus-wide, cross-disciplinary scholarly writing development program supported by a center for biomedical research excellence (COBRE)
Source: PLoS One. 2024 Oct 29;19(10):e0312322. doi: 10.1371/journal.pone.0312322 (PMC11521313; doi:10.1371/journal.pone.0312322)
Supplement: S3 File — (PDF) [file pone.0312322.s003.pdf]

Code System for CCOP Writing Program Evaluation

- Barriers to writing
    - Process Barriers
        - Time management/Lack of time
            - Not scheduling/blocking time to write
            - Not using time scheduled for writing
            - Too many competing priorities
            - Too many interruptions
            - Other/general comment
        - No hard deadline/difficult to prioritize
        - Lack of knowledge about writing
        - Lack of writing experience
        - Difficulty starting writing project
        - Other/general comment
    - People Barriers
        - Lack of collaborators
        - Lack of mentors
        - Lack of people to give feedback on my writing
        - Lack of biostatistics support
        - Other/general comment
    - Internal/Emotional Barriers
        - Lack of motivation/energy to write
        - Lack of confidence
        - Stress related to writing
        - Need for perfection
        - Fear of rejection
        - Imposter Syndrome
        - Other/general comment
    - Lack of incentives
    - Other/general comment

- Writing program elements that were beneficial
    - Accountability measures
        - Submitting word counts
        - Setting timelines/deadlines
        - Setting small/interim/writing session goals
        - Setting retreat goals
        - Setting overall WB 6-month goals
        - Other/general comment
    - Scheduling time to write
    - Group meetings/check ins
        - Getting to know others
        - Learning about other people's research
        - Received peer review or feedback on writing
        - Received advice/suggestions from others/different perspectives
        - Hearing others' successes & challenges
        - Other/general comment
    - Parallel writing/writing alongside others
        - Dedicated/focused/protected writing time
        - Reduced distractions
        - Other/general comment
    - Wellness activities
        - Recharge sessions in W&R
        - Physical environment/retreat setting
        - Leisure activities at retreat
        - Other/general comment
    - How time was used

        - No didactic seminar
        - Participants set their own agenda
        - Other/general comment
    - Competition among participants
    - Other/general comment

- **How writing program benefited participants (what resulted)**
    - Productivity
        - Meeting writing goals
        - Burst of progress/jump start to writing
        - Other/general comment
    - Writing habits
        - Increased or more regular writing
        - Formed new writing partnership or group
        - Other/general comment
    - Improved motivation
    - Better writing product
    - Community of writers
        - Networking/formed new acquaintance
        - Identified new collaborator
        - Started or planned new research/writing project (old/new collaborator)
        - Identified mentor or person willing to provide feedback
        - Learned tips/habits from others
        - Experienced camaraderie among participants
        - Experienced psychological safety/support during discussions
        - Other/general comment
    - Emotions Related to Writing
        - Positive emotions enhanced
            - More enjoyable/fulfilling
            - Increased focus
            - Writing felt easier
            - Increased satisfaction
            - Increased confidence
            - Energized
            - Other/general comment
        - Negative emotions alleviated
            - Less insecurity
            - Less impatience with tedious part of writing
            - Less anxiety
            - Less imposter syndrome
            - Less disappointed in self
            - Not letting others down
            - Other/general comment

- **How writing program could be improved**
    - More group writing
    - Improved accountability
        - Different measures (e.g., word count)
        - Different timing (e.g., weekly log entry)
        - Other/general comment
    - Peer review of writing
    - Other/general comment
    - Expectations were met
    - No changes needed
