# Supplementary material for: First-year evaluation of a campus-wide, cross-disciplinary scholarly writing development program supported by a center for biomedical research excellence (COBRE)
Source: PLoS One. 2024 Oct 29;19(10):e0312322. doi: 10.1371/journal.pone.0312322 (PMC11521313; doi:10.1371/journal.pone.0312322)
Supplement: S4 File — (PDF) [file pone.0312322.s004.pdf]

Consolidated criteria for reporting qualitative studies (COREQ): 32-item checklist
Manuscript title: First-year evaluation of a campus-wide, cross-disciplinary scholarly writing development program supported by a Center for Biomedical Research Excellence (COBRE). (Franks AM et al.)

**Domain 1: Research Team and Reflexivity**	
1. Interviewer/facilitator: Which author(s) conducted the interview or focus groups?	One researcher (BST) conducted all interviews with participants.
2. Credentials: What were the researchers' credentials?	Two researchers (AMF, BST) have extensive experience in conducting qualitative researcher. Three additional researchers (RDL, IK, JW) have extensive experience in study design and evaluation.
3. Occupation: What was their occupation at the time of the stud?	BST is associate professor in the Department of Pharmacy Practice at the University of Arkansas for Medical Sciences College of Pharmacy.
4. Gender: Was the researcher male or female	BST (interviewer) is male. AMF, PD, MA, and JW are female. RDL and IK are male.
5. Experience and training: What experience or training did the researcher have?	AMF and BST have extensive research in conducing qualitative studies.
6. Relationship established: Was a relationship established prior to study commencement?	The interviewer (BST) had no relationship with the participant group, while the broader research team had varying relationships. It was important that BST was seen by participants as having no involvement with the writing program so that they could speak freely about their experiences and opinions. AMF directed the scholarly writing development program that all participants completed and interacted regularly with participants. RDL, IK, MA, and JW had interacted with some participants in the course of their research. PD had no relationships with participants.
7. Participant knowledge of the interviewer: What did the participants know about the researcher?	The interviewer introduced himself to participants as an objective party. He assured participants that the interviews were being conducted for quality improvement purposes and answered questions as they arose.
8. Interviewer characteristics: What characteristics were reported about the interviewer?	The interviewer was not involved in the design or delivery of the scholarly writing development program. He was aware of the general design of the program and participated in the development of the interview questions. No interviewer-related biases were identified.
**Domain 2: Study Design**	
9. Methodological orientation and theory: What methodological orientation was stated to underpin the study?	Content analysis was conducted using constant comparison. The analysis was underpinned by self-determination theory.
10. Sampling: How were participants selected?	Purposive sampling was used.
11. Method of approach: How were participants approached?	Participants were asked to participant in interviews by email and by face-to-face invitations offered during writing program events.
12. Sample size: How many participants were in the study?	14
13. Non-participation: How many people refused to participate or dropped out? Reasons?	Although they did not explicitly refuse participation, 11 participants in the scholarly writing development program did not respond to the invitation to participate in interviews.

14. Setting of data collection: Where was data collected?	Interviews were conducted in one of 2 settings: 1) in person, in a private room during the off-campus overnight writing retreat; and 2) via videoconference.
15. Presence of non-participants: Was anyone else present besides participants and researchers?	No
16. Description of sample: What are the important characteristics of the sample?	All characteristics of the sample are reported in Table 2. Participants consisted of faculty of all academic ranks and staff, with appointments / positions within all colleges of the university. Six (43%) of participants had an affiliation with the CCOP, and 8 (57%) did not
17. Interview guide: Were questions, prompts, guides provided by the authors? Was it pilot tested?	The interview guide included open-ended prompts to solicit information. Questions were developed *a priori* to probe participants' perspectives as well as generated in response to participants' previous answers to elicit greater detail.
18. Repeat interviews: Were repeat interviews carried out?	No
19. Audio/visual recording: Did the research use audio or visual recording to collect the data?	All interviews were audio recorded.
20. Field notes: Were field notes made during and/or after the interview or focus group?	No
21. Duration: What was the duration of the interviews or focus group?	The average duration of interview was ~28 minutes (17-39 minutes).
22. Data saturation: Was data saturation discussed?	Interviews were continued until data saturation was reached.
23. Transcripts returned: Were transcripts returned to participants for comment and/or correction?	No
**Domain 3: Analysis and Findings**	
24. Number of data coders: How many data coders coded the data?	Two researchers (AMF and PD) independently coded the data. A third researcher (BST) was available to resolve discrepancies in coding as needed.
25. Description of the coding tree: Did authors provide a description of the coding tree?	The description of the coding scheme is provided Methods section, Evaluation sub-section.
26. Derivation of themes: Were themes identified in advance or derived from the data?	Themes were derived from the data.
27. Software: What software, if applicable, was used to manage the data?	MaxQDA 2020 was used.
28. Participant checking: Did participants provide feedback on the findings?	No
29. Quotations presented: Were participant quotations presented to illustrate the	Yes. Representative quotations are presented in Tables 3, 4, and 5. Each quotation is attributed to a participant using anonymized participant numbers.

themes / findings? Was each quotation identified?	
30. Data and findings consistent: Was there consistency between the data presented and the findings?	Yes. The themes derived from the data are consistent with the original data.
31. Clarity of major themes: Were major themes clearly presented in the findings?	Yes.
32. Clarity of minor themes: Is there a description of diverse cases or discussion of minor themes?	Minor themes are not explicitly included. The Results section includes description of diverse cases (e.g., suggested improvements to the scholarly writing development program) to reflect the varying perspectives and opinions of participants.
